# Extrapulmonary Small Cell Carcinoma Presenting as an Orbital Mass: A Case Report

**DOI:** 10.7759/cureus.26012

**Published:** 2022-06-16

**Authors:** Anna-Lena Meinhardt, Aditya Uppuluri, Elina Shkolnik, Victor T Chang

**Affiliations:** 1 Internal Medicine, Rutgers University New Jersey Medical School, Newark, USA; 2 Ophthalmology, Rutgers University New Jersey Medical School, Newark, USA; 3 Pathology and Laboratory Medicine, Rutgers University New Jersey Medical School, Newark, USA; 4 Hematology and Oncology, East Orange Veterans Affairs Medical Center, East Orange, USA; 5 Hematology and Oncology, Rutgers University New Jersey Medical School, Newark, USA

**Keywords:** small cell carcinoma, orbital malignancy, episcleritis, neuroendocrine neoplasm, intra orbital mass, extrapulmonary small cell carcinoma

## Abstract

Extrapulmonary small cell carcinomas (ESCCs) are poorly differentiated neuroendocrine tumors that are characterized by an aggressive course and poor survival rates. While these tumors can be found anywhere in the body, presentations of lesions in the orbit are exceedingly rare. We present the case of a 47-year-old man who presented with blurry vision, lacrimation, and tenderness of his right eye, as well as a small but palpable temporal mass. Upon workup, he was diagnosed with ESCC in the orbit as well as lesions in the liver and spine. He received systemic chemotherapy but unfortunately proceeded to have rapid spread of his disease and succumbed to this cancer only a year after presentation. This patient illustrates the importance of developing optimal treatment strategies, which have yet to be delineated, and especially the impact of newer immunotherapy agents remains to be seen.

## Introduction

Small cell carcinomas are poorly differentiated neuroendocrine tumors that are most commonly seen in the lung. In the 1930s, these tumors were also discovered outside of the lung, and since then extrapulmonary small cell carcinomas (ESCCs) have been found in almost every organ system. Most commonly they are found in the gastrointestinal (GI) tract, bladder, prostate, larynx, and the salivary gland [1]. They were long thought to originate from cells of the amine precursor uptake and decarboxylation system [2], but other more recent theories propose that they arise from a multipotent stem cell [3]. The diagnosis requires both the presence of typical pathologic features of small cell carcinoma, as well as the absence of pulmonary disease in imaging studies [4]. Overall, ESCCs remain relatively rare malignancies, and therefore their management has been based on that of small cell lung cancer, given their similar cells of origin [2]. The five-year overall survival of patients is dependent on the tumor site but remains low and ranges between 2.0% and 43% [5]. Here, we present a rare case of orbital extrapulmonary small cell cancer, of which only a handful have been reported in the literature.

## Case presentation

A 47-year-old man presented to the optometry clinic with blurry vision, as well as one day of tearing and irritation in the right eye. He also noted mild tenderness of the right eyelid, mild facial pain above the right eye at severity of 3/10, and a four-day history of a painful temporal bump. The patient did not have a history of any ophthalmic or sinus disease. His past medical history included hypertension, polysubstance abuse, untreated hepatitis C, depression, and tobacco use for 30 pack-years. Physical examination demonstrated mild swelling of the right upper eyelid, mild corneal dystrophy, and unilateral conjunctival hyperemia. The initial impression was episcleritis, so he was started on fluorometholone and advised to return to the clinic one week later.

**Figure 1:**
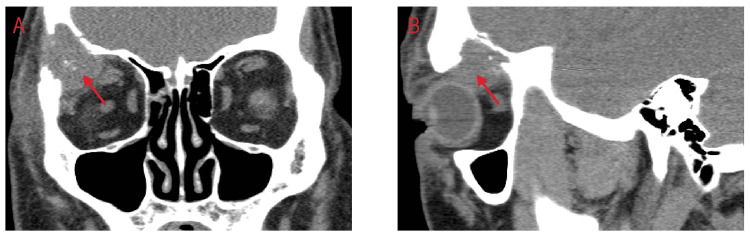
CT imaging of the orbits. (A) Coronal and (B) sagittal views showing a lytic lesion with a soft tissue mass in the lateral wall and roof of the right orbit with a mass effect on the superior and lateral rectus muscles and on the right globe.

However, the next day he returned with an inability to open his right eye and worsening pain, especially when moving his eye, as well as swelling, tearing, and itching of the right eye. He reported new diplopia on the upward and right gaze with restriction in eye movements. Intraocular pressure had increased to 26 mmHg from 22 mmHg the day prior. On examination, he had a 50% reduction of upward gaze, 90% of right gaze, 2-3+ edema of right upper and lower eyelids, with tenderness, and right eye chemosis. CT imaging of the orbits showed a lytic lesion with a soft tissue mass in the lateral wall and roof of the right orbit with a mass effect on the superior and lateral rectus muscles and on the right globe. This was suspected to be a metastatic lesion or a primary bone neoplasm such as plasmacytoma (Figure 1). MRI could not be performed on this patient. Clinically he was felt to have periorbital cellulitis and inflammation from the underlying tumor.

**Figure 2:**
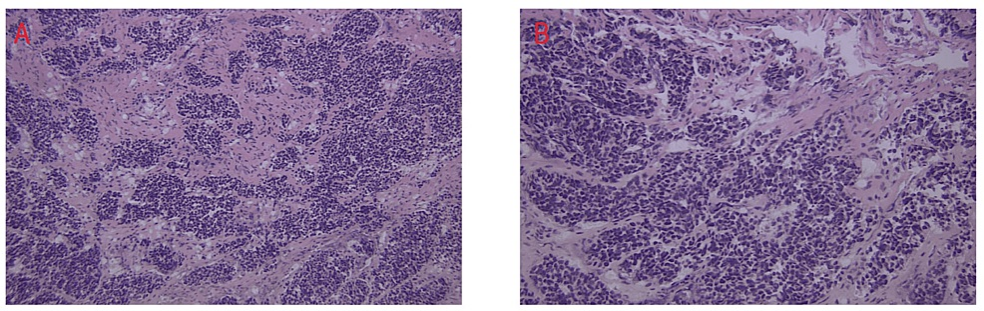
H&E stain of the periorbital tumor. Hematoxylin-eosin (H&E) stain of the periorbital tumor at (A) 20x magnification and (B) 40x magnification demonstrating small- to intermediate-sized, round-to-oval cells with scant cytoplasm.

**Figure 3:**
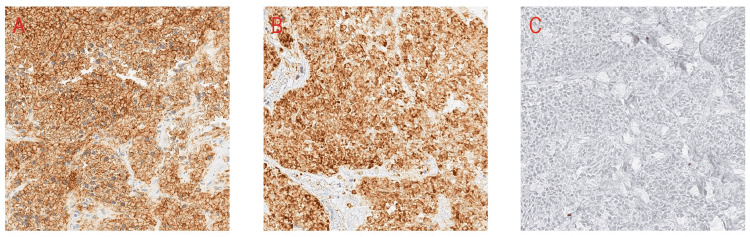
Immunohistochemical stains of the periorbital tumor. Immunohistochemical (IHC) stains were positive for CD56 (A) and synaptophysin (B) and negative for CK5/6 (C). Other stains are not shown.

A biopsy of the periorbital tumor was performed and it revealed extrapulmonary small cell neuroendocrine carcinoma in the lacrimal gland with necrosis and bone invasion (Figure 2). Immunohistochemical studies showed immunoreactivity to synaptophysin, CD56 (Figure 3), and MCK, and were negative for NSE, chromogranin, TTF-1, CK 5/6, HMB 45, B-100, vimentin, desmin MyoD1, Flt1-1, CD99, and CD45. Further workup included CT imaging of the chest, abdomen, and pelvis, which demonstrated liver nodules, as well as multiple enlarged iliac, precaval, inguinal, and celiac lymph nodes. Positron emission tomography (PET)/CT scan confirmed an enhancing soft-tissue lesion within the superior aspect of the lateral and right orbital wall and metastases in the liver and spine. At this point, he was diagnosed with ESCC metastatic to multiple sites, and only the lungs as the primary site could be excluded. One month after the initial presentation he was therefore started on chemotherapy with cisplatin and etoposide.

Three months after initiation of chemotherapy, CT imaging showed a new blastic lesion of the left iliac bone. He was started on palliative radiation for this lesion. Five months later, the patient presented with shoulder, axillary, and chest pain, and MRI showed new metastases to numerous vertebral bodies with mass effect over the right lateral aspect of the spinal cord at T2. Palliative radiation to the thoracic spine was started. The next month, even more vertebral lesions as well as a splenic lesion were found, which was followed by additional radiotherapy. Starting of topotecan was discussed; however, the veteran decided against further chemotherapy. Twelve months after his initial presentation, the patient was again admitted to our hospital for worsening back pain secondary to bone metastases, and the plan was made to discharge him to home hospice. Unfortunately, while awaiting arrangements, the patient died.

## Discussion

ESCCs are rare, have an incidence rate of about 0.1-0.4%, and are most often described in the GI tract, bladder, prostate, larynx, and salivary gland [1]. The orbit as the primary presenting site for ESCC tumor presentation or metastasis is more uncommon, and there have been only a few reported cases in the literature. While metastatic disease to the orbit is relatively rare, it does occur in 2-3% of patients with systemic cancer, and it makes up 1-13% of all orbital tumors [6]. The most common cancer types to metastasize to the orbit are breast and prostate cancer, but melanoma, neuroendocrine tumors, and tumors of the GI tract have been described as well [6]. In our case, we were able to rule out lung cancer, rhabdomyosarcoma, Ewing’s sarcoma, and skin malignancies based on immunohistochemistry, and could classify the malignancy as a neuroendocrine tumor. Establishing a primary site was challenging, and possibilities included unknown primary, liver ESCC metastatic to the orbit, or primary orbital ESCC metastatic to the liver. Small cell carcinomas of the orbit are considered a rare entity. They are often the result of metastasis from a distant site [7], local invasion from a small cell carcinoma of the sinuses [8], and only rarely a primary malignancy of the orbit [9].

The most common symptoms of any kind of orbital tumor upon presentation are proptosis, periorbital pain, orbital swelling, and diplopia, as well as restriction of extraocular movements [10]. Orbital ESCCs tend to present very similarly to more common and non-neoplastic orbital pathology such as orbital abscess [9] or episcleritis as seen in the present case. Given the paucity of cases, it is difficult to establish clear gender predilection; however, the few reported cases show a female predilection with a median age of diagnosis in the 40s [9]. Specifically, in the case of ESCC, symptom onset seems to be rapid and within days to weeks as was the case in our patient [9]. Making a definite diagnosis as quickly as possible is vital given the speedy progression and poor prognosis of this neoplasm. The challenge lies not only in diagnosing the malignancy histologically but also in performing systemic staging given the high likelihood of other organ involvement at the time of diagnosis. While CT and MRI are often considered the standard imaging techniques, PET/CT imaging has also emerged as a valuable tool in diagnosis, biopsy planning, and staging [11]. Distinguishing orbital ESCC from sinonasal ESCC is another important aspect. While the latter can cause more distinctive complaints such as epistaxis and nasal obstruction, it also often merely presents with vague symptoms of facial pain and exophthalmos [12]. However, both of these entities remain rare findings, and standardized approaches have not yet been established for either case.

Efficient collaboration among ophthalmologists, oncologists, radiologists, and pathologists is necessary for prompt evaluation and initiation of treatment. This primarily consists of chemotherapy, radiation therapy, surgical excision, and, more recently, immunotherapy. Pain management is another important aspect of treatment and preservation of quality of life, and can be achieved through medications or radiation of bone metastasis. Non-medical supportive care is also of utmost importance as patients have to cope with the acute and rapidly progressing loss of vision, as well as possible cosmetic and psychosocial consequences of an orbital tumor. While the survival of orbital ESCC tends to be poor and is less than two years from the time of diagnosis [9], the advance in immunotherapy in treating pulmonary small cell carcinoma could highlight a new approach to treat ESCC as well. Individual cases where immunotherapy was used with promising results in ESCC have been published [13,14], indicating that the role of these new medications in treating ESCC should be further investigated. Additionally, radiation of the orbit has been effective in preserving vision in the case of orbital metastases of other cancer types and may be useful in ESCC as well [15].

## Conclusions

In conclusion, we want to emphasize that while orbital ESCC is a rare presentation, it is a diagnosis that should be considered in any patient presenting with persistent or progressive, unexplained symptoms or any orbital mass found on imaging.
